# Alginate-Based Electrospun Nanofibers and the Enabled Drug Controlled Release Profiles: A Review

**DOI:** 10.3390/biom14070789

**Published:** 2024-07-03

**Authors:** Zhiyuan Zhang, Hui Liu, Deng-Guang Yu, Sim-Wan Annie Bligh

**Affiliations:** 1 School of Materials and Chemistry, University of Shanghai for Science and Technology, Shanghai 200093, China; 223353170@st.usst.edu.cn (Z.Z.); huiliu@usst.edu.cn (H.L.); 2 School of Health Sciences, Saint Francis University, Hong Kong 999077, China

**Keywords:** alginate, electrospinning, biomedical, nanostructure, drug delivery, controlled release

## Abstract

Alginate is a natural polymer with good biocompatible properties and is a potential polymeric material for the sustainable development and replacement of petroleum derivatives. However, the non-spinnability of pure alginate solutions has hindered the expansion of alginate applications. With the continuous development of electrospinning technology, synthetic polymers, such as PEO and PVA, are used as co-spinning agents to increase the spinnability of alginate. Moreover, the coaxial, parallel Janus, tertiary and other diverse and novel electrospun fiber structures prepared by multi-fluid electrospinning have found a new breakthrough for the problem of poor spinning of natural polymers. Meanwhile, the diverse electrospun fiber structures effectively achieve multiple release modes of drugs. The powerful combination of alginate and electrostatic spinning is widely used in many biomedical fields, such as tissue engineering, regenerative engineering, bioscaffolds, and drug delivery, and the research fever continues to climb. This is particularly true for the controlled delivery aspect of drugs. This review provides a brief overview of alginate, introduces new advances in electrostatic spinning, and highlights the research progress of alginate-based electrospun nanofibers in achieving various controlled release modes, such as pulsed release, sustained release, biphasic release, responsive release, and targeted release.

## 1. Introduction

Sodium alginate (SA) is a natural polymer extracted from marine algae that has a wide range of applications in the industrial field because of its unique and excellent structural and chemical properties, including the preparation of new materials for pharmaceutical preparations, the biomedical field, the food field, the cosmetic field, and the environment field [1,2,3,4,5,6]. Natural reserves of SA are very abundant, and natural polymers are preferred over synthetic polymers due to their excellent biocompatibility and natural degradability, and they are considered sustainable and promising alternatives to petroleum-derived polymers [7,8].

With the development of nanotechnology, the preparation of nanostructured SA materials has gained in-depth research and made great progress [9]. Nowadays, multiple techniques are available to prepare alginate-based nanomaterials with different sizes, morphologies, and compositions, such as electrostatic spinning [10], electrostatic spraying [11], controlled gelation [12], 3D bioprinting [13], cast film [14], self-assembly [15], phase separation [16], microfluidic technology [17], and other technologies. Electrostatic spinning technology is a cutting-edge, convenient, cost-effective, and commonly used preparation method for preparing nanofibers. In the past decades, researchers have deeply explored the biomedical applications of electrospun alginate-based nanofibers in tissue engineering, regenerative engineering, bioscaffolds, and drug and gene delivery [3,10,18,19,20]. In order to achieve and maximize the desired therapeutic effect, the drug needs to be embedded in a suitable delivery system so as to achieve specific release characteristics [21]. Among them, electrospun nanofibers are considered one of the most promising nanotechnologies for overcoming the current challenges and shortcomings of modern drug delivery systems and achieving the desired release pattern [22,23,24]. Sodium alginate nanofiber (SANF) membranes prepared based on electrostatic spinning technology have excellent properties, such as high specific surface area, high drug loading capacity, controlled drug delivery, and reduced drug side effects [21]. In addition, the nanomaterials transformed by electrospun alginate nanofibers (EANFs) possess unique properties, such as the balance between degradation rate and swelling index, which greatly facilitates the development of new-generation scaffolds, drug delivery systems, and regenerative biomaterials [25].

However, the poor spinnability of natural polymers is a non-negligible problem [26,27]. Synthetic polymers are superior to natural polymers in terms of processability, mechanical properties, loading properties, and reusability. On the other hand, synthetic polymers are cost effective as carriers. However, synthetic polymers are inert, do not adequately interact with surrounding cells in most cases [3], and are not easily degraded [28]. The combination of natural and synthetic polymers for the development of electrospun delivery drug systems is an ingenious strategy to address the issues of functionality, biocompatibility, and material–tissue integration [3]. Meanwhile, nanofibers with different structures prepared by single-fluid, two-fluid, and multi-fluid electrospinning can improve the spinnability of spinning dysfunctional polymers to some extent [26,29,30].

**Figure 1:**
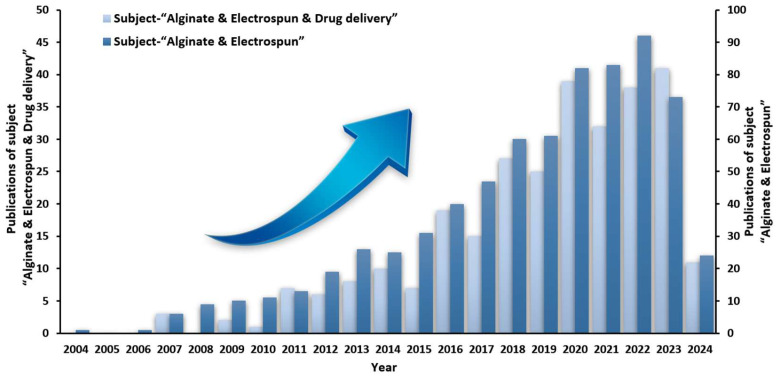
Statistics of literature retrieval on the “Web of Science” platform with the subjects of “Alginate & Electrospinning” and “Alginate & Electrospinning & drug delivery”.

The literature on “electrospinning & alginate” and “electrospinning & alginate & drug delivery” was searched on the “Web of Science” platform from 2004 to 2024. As shown in Figure 1, the amount of literature on the topic of “electrospinning & alginate” has been increasing year by year, indicating that the preparation of SANFs by electrospinning has become a research hotspot. Although the number of papers with the theme of “electrospinning & alginate & drug delivery” is small, it is also on the rise, indicating that the development prospect of electrospun nanofibers in the field of drug delivery is also very bright.

**Figure 2:**
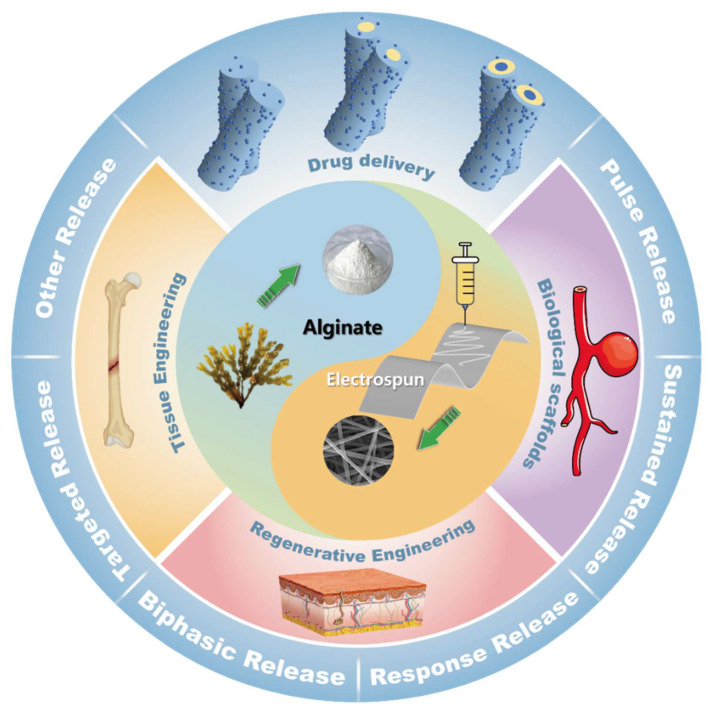
Alginate electrospun fibers for biomedical applications and for drug delivery and controlled release modalities.

This paper reviews the development of alginate and electrostatic spinning technologies, and introduces the preparation of electrospun fibers with different structures by single-fluid, two-fluid, and multi-fluid. The electrospun alginate drug delivery system (EADDS) is also used to realize a variety of controlled release modes for different drugs, such as pulsatile release, sustained release, biphasic release, responsive release, and targeted release, to meet applications in various fields (Figure 2).

## 2. Alginate

Alginates are a class of naturally occurring linear anionic polysaccharides that are mainly found in the cell walls and intercellular mucilages of brown algae, but also in some mucilage-producing pods of bacteria, such as *Pseudomonas aeruginosa* and nitrogen-fixing bacteria [31]. SA exists in the intracellular matrix of algae in the form of a gel containing ions, which are used to maintain ion exchange between the algae and the external seawater environment. Ions in the gel form include sodium, calcium, magnesium, strontium, copper, and barium ions. The process of SA synthesis includes biosynthesis and artificial extraction [1]. Biosynthesis is the conversion of GDP-mannuronic acid precursors from carbon sources in bacteria, which undergo polymerization, transfer, and modification and are eventually converted to SA for excretion from the outer membrane. Artificial processes include removal of counterions, removal of particulate matter, drying, and grinding to extract. Free-flow electrophoresis was used to remove mitogen and cytotoxic impurities. SA was synthesized and extracted in a manner that demonstrated its wide availability, ease of handling, and low cost.

**Figure 3:**
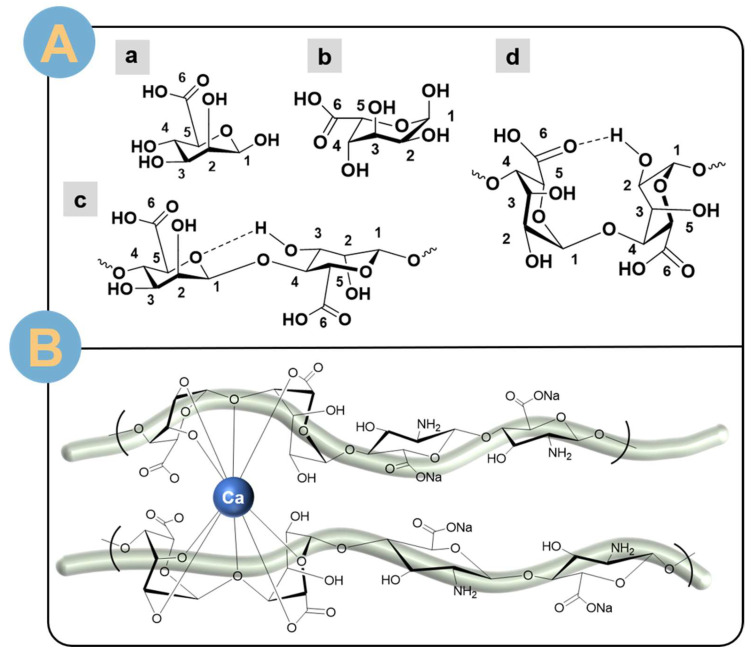
(**A**) Structures of alginate epimeric blocks: (**a**) β-d-mannuronic acid (M block) and (**b**) α-l-guluronic acid (G block) as well as homopolymer blocks; (**c**) MM block (mannuronic acid) and (**d**) GG block (guluronic acid). (**B**) Schematic diagram of CaCl_2_ cross-linked SA.

The structural type of SA depends on multiple combinations of two monomer blocks. The monomeric units of SA are two sugar epimers, β-D-mannuronic (M) (Figure 3A(a)) and α-L-guluronic (G) (Figure 3A(b), which are covalently linked to each other via 1–4 glycosidic bonds (Figure 3A(c,d)) and form polyguluronic acid (GG…) (Figure 3A(d)) and polymannuronic acid (MM…) (Figure 3A(c)) homopolymerized blocks [32]. The GG block has a more rigid structural conformation relative to MM, which has a linear structure. The strong rigidity is due to the formation of hydrogen bonds facilitating the transition to a “flexural“ conformation between the hydroxyl group of one residue and the carboxylic acid group of the neighboring residue [32]. SA has several different fragments throughout the molecular chain: (G-G)_n_, (G-M)_n_, and (M-M)_n_. The high content of the G block is a generalization originating from algal alginates, a property that makes SA uniquely advantageous for biomedical applications. Alginates of bacterial origin are enriched in M-blocks, such as SA produced by *Azotobacter* and *Pseudomonas* through microbial enzymatic degradation [33]. SA enriched with group M blocks is immunogenic and can effectively induce cytokine production [34].

Although SA is insoluble in organic solutions, such as ethanol, chloroform, and ether, it is more soluble in water and alkaline solutions [1]. The difference in solubility is affected by the pH of the solvent in addition to the ionic strength of the medium, and the gel ions in the solvent likewise cause changes in the solubility of SA. The pH of the solution is not lower than the critical pH, and deprotonation of carboxylic acid groups is necessary for SA solubility. The ionic strength of the medium causes changes in the structural behavior, chain length, and viscosity of the solute, and the solubility varies. In addition, the presence of divalent cations promotes the insolubilization of SA, causing it to form gels, such as sodium, calcium, magnesium, and strontium ions. SA has a strong gel-forming ability due to the fact that Na^+^ on the G block can react with other divalent cations, and when Na+ is lost on the G block, the molecules are deposited, which cross-links to form a three-dimensional mesh structure [35,36]. The introduction of calcium ions causes the G-block of SA to be tightly connected to each other (Figure 3B), building a “pearl-box” structure [37]. Calcium ions can attract the O atoms of the G block on the same SA chain or on different SA chains to chelate and promote SA gelation. Therefore, G-block-rich SAs will form gels with stronger structural properties. Meanwhile, divalent Pb ions have the strongest affinity for SA relative to copper, cadmium, barium, strontium, and calcium ions. Divalent calcium ions are the most widely used in the gelation of SA.

SA is an ECM-like natural material with a structure similar to the extracellular matrix (ECM) of living tissues [3]. In addition, SA is low-cost, easily accessible, non-toxic, antimicrobial, highly biocompatible, and hygroscopic, and it has great potential for use in regenerative engineering and drug delivery [3,10,38,39].

The SA derivatives obtained by modification of SA have properties more in line with expectations and a wider range of applications. Solubility is one of the important factors in the modification of SA [1]. The SA may first be dissolved in its soluble solvent or solvent system and then modified. In addition, the reactivity of SA is a determining factor in the treatment of alginate modification. Two types of reactive sites, sec-OH sites (C-2 and C-3) or one -COOH site (C-6), are the main starting points for the reduction of SA [1]. The two types of functional groups react differently, making selective modification possible. For oxidation, sulfation, and esterification modification of hydroxyl groups of alginate skeleton, the protons of hydroxyl groups are replaced by new atoms or functional groups that lack the ability to form hydrogen bonds [9,40]. Moreover, it has been shown that modification of the alginate backbone prolongs the release of the transforming growth factor [41]. On the other hand, M or G residues were selectively modified, such as by utilizing the selective chelation of G residues in alginate Ca gels, which could control the reaction [37]. It is worth noting that the reactivity of SA to acids, bases, and reducing agents has an effect on the modification, and competition for the degradation reaction rapidly reduces the molecular weight. The alginate backbone was modified to reduce the hydrogen bond density, greatly improving the electrospinning properties [9].

## 3. Electrospinning

### 3.1. Introduction of Electrospinning

Electrostatic spinning, as a bottom-up nanomaterial preparation technique, can process precursors into continuous nanofibers as fine as 2 nm and as coarse as a few micrometers [42]. A representative electrostatic spinning device, spatially positioned from top to bottom, consists of several parts: an axial flow syringe pump, a syringe (usually containing solvent and equipped with a stainless steel spinning head at the front end), a DC high-voltage generator, and a grounded collector plate (usually an aluminum foil) [43]. An electrode of the high-voltage generator is connected to the metal of the spinning head at the front end of the injector to energize the precursor by charge conduction. The electrospinning precursors are mainly polymer solutions, but melts can also be prepared by electrospinning into continuous fibers [44]. The polymer solution or melt is flowed and deformed by a high-voltage electric field, which produces a Taylor cone at the tip of the spinneret. When the electric field is strong enough to cause the droplets to overcome surface tension, they extend into a high-speed jet of uniform thickness. Bending instability causes the jet filaments to produce whipping motion in the medium and also filament splitting [43]. The external medium for jet filament movement is usually air, but in wet spinning, the spinning device is immersed in chemicals that selectively retain the nanofibers through dilution effects or chemical reactions. The jet filaments are removed by evaporation of the solvent or by the reaction, and the retained cured homogeneous fibers are deposited on the receiving device to form a two-dimensional nanofiber film. The receiving device is adapted to obtain fibers that are orientationally arranged in a clockwise order [45], and asterisk-like fibers [46]. In addition, the receiving device is modified into a hemisphere with an internally arranged metal needle array, which can directly transform one-dimensional nanofibers into a three-dimensional sponge structure with porous and loosely arranged fibers [47].

Electrostatic spinning technology has been widely studied thanks to the advantages of simple spinning device, low cost, and controllable process [42,48]. From the open literature, it was found that by varying the reaction parameters such as relative molecular mass, solution viscosity, and applied voltage, the electrospun fibers now have a size window of up to 1 nm [49] and a few micrometers for the coarse ones [42]. Adjusting the electrospinning parameters, such as feed rate, voltage intensity, and receiving distance, researchers usually prepare single polymer fibers with uniform diameter, smooth surface, and regular round cross-section. As the research progresses, the advantages of rough surface and bead string structures of fibers are being discovered [50,51]. In order to broaden the effective application of electrospun nanofibers in different scenarios, more and more interesting electrospun polymer fibers with tunable secondary structures are being designed and prepared.

There is a wide variety of polymers for electrospinning, mainly including synthetic polymers, naturally derived polymers, multi-component composite polymers, and inorganic/organic composites. Among the synthetic polymers, the commonly used synthetic biopolymers are poly(ε-caprolactone) (PCL), poly(lactic acid) (PLA), poly(lactic-co-glycolic acid) (PLGA), poly(ethylene glycol)-copolymerized (d,l-lactone) (PELA), poly(ethylene glycol)-poly(ε-caprolactone) (PEG-PCL), polystyrene (PS), polyvinyl alcohol (PVA), polyvinyl pyrrolidone (PVP), and polyethylene oxide (PEO); natural polymers can be categorized into polysaccharide-based materials and protein- or peptide-based materials [52,53,54], which are better used in living organisms due to their better biocompatibility and non-toxicity. Some of the commonly used natural polymers are SA, chitosan (CS), cyclodextrin, gelatin, collagen, silk fibroin, and spider silk protein. It should not be overlooked that many natural polymers are characterized by poor solubility and low molecular weight, making it difficult for them to be directly electrospun. The strategy of co-spinning synthetic and natural polymers not only compensates for the poor biocompatibility of synthetic polymers but also improves the spinnability of natural polymers. Polylactic acid, PLGA, PCL, PEO, or PVA are often used as carrier polymers for co-electrospinning with natural polymers [25,55,56]. On the other hand, the internal structure of a single electrospun fiber likewise increases the spinnability of natural polymers and enables purposeful control of the loading substance. Electrospun fibers are used in wound dressings, food, drug delivery, piezoelectric catalysis, photocatalysis, and sensors [56,57,58,59,60,61,62,63].

### 3.2. Diverse Electrospun Fiber Structures

#### 3.2.1. Single Fluid Electrospinning

**Figure 4:**
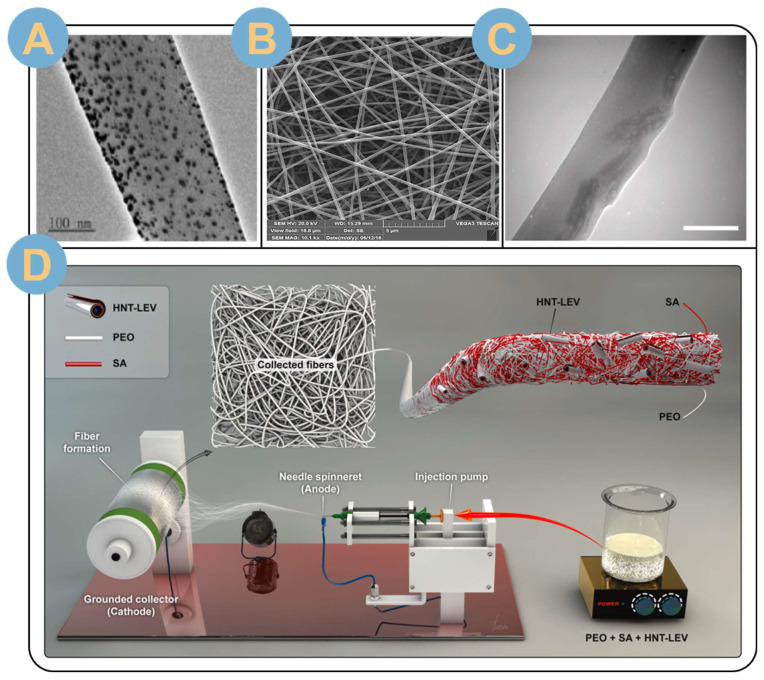
(**A**) TEM image of embedded nanosilver PCL fibers. (scale = 100 nm) [20]. (**B**) SEM images of 0.2% (*w*/*v*) collagen grafted nanofiber [74]. (**C**) TEM image of HNT-LEV/SA-PEO nanocomposite fibers at an electron acceleration voltage of 100 kV (scale = 300 nm) [65]. (**D**) Schematic diagram of the research-designed electrospinning process (two-column diagram) [65].

Single-fluid electrospinning is the most widely studied due to its simplicity and ease of preparation, which can be direct electrospinning of a single polymer dissolved in a solvent. Single electrospun nanofibers were empowered by mixing inorganic nanoparticles, nanotubes, or drugs in the electrospinning precursor solution [64,65,66,67]. It was shown that the metal nanoparticles introduced into the electrospinning precursor could be uniformly distributed inside PCL nanofibers with a size of 172.2 ± 48.9 nm (Figure 4A) [20]. Moreover, the electrospinning process does not cause morphological changes in the metal nanoparticle fillers [68]. Dodero et al. [69] successfully embedded ZnO nanoparticles in alginate nanofibers by single-fluid electrospinning. The diameter of the nanofibers was uniform, with an average diameter of about 100 nm and an average inter-fiber gap of about 140 nm. In another study, Dodero et al. [70] successfully introduced ZnO nanoparticles on alginate-based mats and formed a thin and uniform nanofiber membrane of 100 ± 30 nm after washing-crosslinking. The embedding of zinc oxide nanoparticles greatly improved the antimicrobial and antibacterial properties and provided a simpler and safer method for the production of surgical patches and wound dressing membranes. In addition to embedding a single metal nanoparticle, the simultaneous embedding of two nanoparticles was also realized by the researchers. Co-electrospinning of combined synthetic and natural polymers can broaden the application of single-fluid electrospun fibers in the fields of tissue engineering, regenerative engineering, and bioscaffolds [71,72,73]. Sobhanian et al. [74] used a mixture of poly(vinyl alcohol), gelatin, and SA as precursors, and employed a single-fluid electrostatic spinning process to nanofibers with a diameter of about 229 nm were prepared (Figure 4B). The nanofibers were uniform and straight, with no bead string structure. Fiber deposition converted into nanofibrous membranes was used as regenerative skin tissue grafted to rat tails. The incorporation of natural polymers results in some loss of tensile strength of the nanofibers, but, more importantly, enhances the biocompatibility of the fibrous membranes and their ability to promote wound healing. Chien et al. [64] embedded both zinc oxide nanoparticles and graphene oxide (GO) nanoparticles in polyvinyl alcohol/sodium alginate (PVA/SA) nanofiber (GO) nanoparticles. The PVA/SA/GO/ZnO nanofibers had a uniform diameter with an average diameter of 199 ± 22 nm. Glutaraldehyde (GA) vapor environment cross-linking welded the junctions between the nanofibers to form porous nanofiber membranes with a mesh structure. The crosslinked hybridized nanofibrous membranes had excellent hemostatic and exudate-absorbing properties, providing a moderately moist environment for wound healing and promoting wound healing. In addition to doping nanoparticles in the spinning solution, it is also possible to mix linkers to promote the formation of nano-nets. Topuz et al. [75] electrospun mixed cyclodextrins (CDs) with graphitic acid linkers in solution monofluidic electrospinning into filaments. Further thermal cross-linking post-treatment resulted in the formation of a hyper-cross-linked mesh structure of CD, a cyclic oligosaccharide with a hydrophobic inner surface, and a hydrophilic outer surface. Molecular modeling demonstrated that cross-linking had little effect on the host–guest interactions of the fibrous CD network. The CD natural eco-friendly fiber membrane prepared by electrospinning-crosslinking showed good adsorption and removal of textile dyes and polycyclic aromatic hydrocarbon (PAH) pollutants, with a maximum adsorption capacity of 692 mg/g. In addition, the CD natural eco-friendly fiber membrane possessed good ultrasound-assisted recyclability, which made it an excellent green and pollution-free material. The uniform loading of nanotubes is also a challenging research direction. sodium alginate (SA)/poly(ethylene oxide) (PEO) nanocomposite fibers prepared by single-fluid electrospinning were successfully embedded with levofloxacin (LEV)-loaded halloysin nanotubes (HNT) by Fatahi et al. [65] (Figure 4D).The HNT-LEV/SA-PEO nanocomposite fiber mats were very homogeneous and did not contain any beads with an average diameter of 330 ± 17 nm. TEM images confirmed that the HNT-LEV nanohybrid particles were embedded in an oriented manner along the fiber direction (Figure 4C) and were well dispersed in the nanofiber matrix without agglomeration.

The simplicity and convenience of single-fluid electrospinning also brings limitations to fiber structure and functionality, which has led researchers to develop and design two- and multi-fluid nanofibers based on single-fluid electrospinning studies [76,77,78].

#### 3.2.2. Double Fluid Electrospinning

Double-fluid electrospinning nanofibers mainly include core-shell structure and side-by-side Janus structure [79,80]. In order to improve the inter-fiber porosity, there are also a few studies on conjugate electrospinning [29]. The core-shell structure has been studied more extensively than the side-by-side Janus structure, but different structures have different advantages [81,82,83]. Both structures formed by two-fluid electrospinning are structural cornerstones for providing superior performance and preparing advanced materials, and many of the more complex structures are essentially combinations or derivatives of the two.

**Figure 5:**
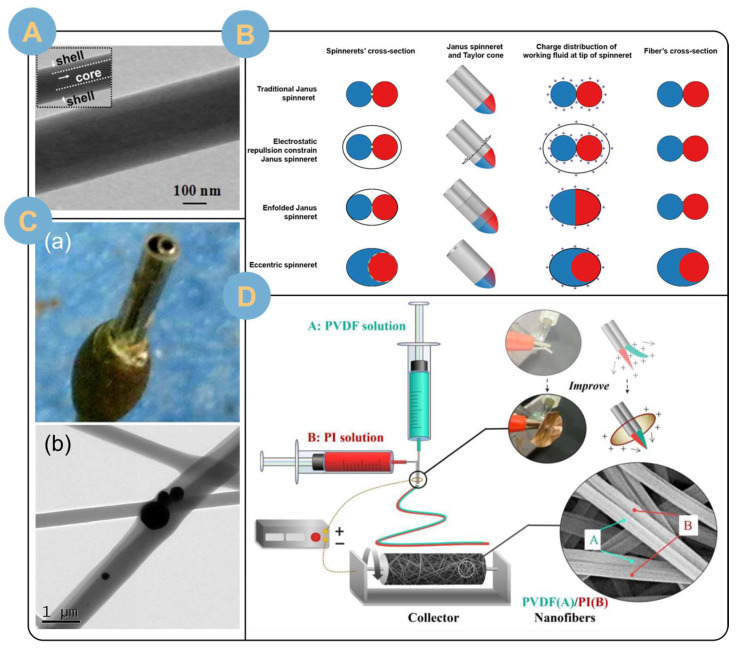
(**A**) TEM images showing the coaxial structure of nanofibers [85]. (**B**) Schematic diagram of the electrospinning of Janus fibers through different spinnerets [29]. (**C**) (**a**) Digital photograph of a concentric side-by-side nozzle design. (**b**) TEM images of Janus-structured electrospun fibers simultaneously loaded with CIPs and AgNPs [87]. (**D**) Design diagram of the preparation of electrospun juxtaposed structured nanofibers [88].

Coaxial electrostatic spinning technology allows two precursors to be encapsulated into filaments in separate nuclear and shell layers through dual channels. The substance in the core layer is not in contact with the external environment, which greatly facilitates the sustained release of the encapsulated drug. Zhu et al. [84] loaded the active pharmaceutical ingredient of cumaru glycoside in CS solution as the core layer and the mixed solution of SA and PVA as the shell layer, and successfully electrospun the coaxial structured nanofibers using coaxial spinning equipment. The core-shell structure nanofibers loaded with *Centella asiatica* glycosides can be observed under a scanning electron microscope, which shows that the surface of the fibers is smooth, the size is uniform, and there are few bead strings. The core-shell structure of the fibers is obvious, with an inner diameter of about 98.1 nm and an outer diameter of about 168.5 nm. In vitro drug release experiments and burn wound healing experiments show that the continuous release of *Centella asiatica* glycosides placed in the core layer leads to the rapid healing of burn wounds. In addition, coaxial electrospinning provides a better platform for targeted drug delivery and release. Feng et al. [85] coaxially electrospun uniform nanofibers with an average diameter of 241 ± 38 nm by using SA as the shell layer and pectin-coated liposomes of a bioactive peptide, salmon calcitonin (sCT), as a core fiber (Figure 5A). The design of the core-shell nanofibers mitigated the burst release of sCT in simulated gastric and intestinal fluids (SGF and SIF), providing a superior multi-unit carrier for the sustained release of orally bioactive peptides or proteins for colon-targeted drug delivery. Chen et al. [86] loaded the drug curcumin (Cur) and the metallic antimicrobial agent zinc oxide (ZnO) into the core and sheath layers, respectively.

Unlike coaxial electrospinning, side-by-side electrostatic spinning can vary the different structures of the spinnerets to regulate the degree of envelopment of the two fluids [29] (Figure 5B). The external wrapped side is referred to as the crescent or curved moon side, and the internal wrapped side is referred to as the rounded side. The degree of wrapping of the internal round side by the external curved moon side influences the area of the round side in contact with the external environmental exposure to control the diffusion rate and release time of the wrapped substance. Yang et al. [87] prepared Janus structured nanofibers using a side-by-side electrospinning process. The curved moon side polyvinylpyrrolidone (PVP) was loaded with the drug ciprofloxacin (CIP), and silver nanoparticles (AgNPs) were blended with ethylcellulose (EC) as a precursor on the round side. Homemade center spinnerets (Figure 5C(a)) collected continuous Janus-structured nanofibers with an average diameter of 0.78 ± 0.18 μm. The juxtaposed Janus structures of the fibers were presented clearly on the SEM images (Figure 5C(b)), and the AgNPs were clearly distributed on the curved moon side. In addition, the drug CIPs loaded on the circular EC side were completely transformed into an amorphous state distribution. On the other hand, the adhesion force of the solution between the two fluids could drive the electrostatic spinning of non-spinnable substances, especially for dilute fluids, to improve the spinnability [29]. However, it is worth noting that the preparation of juxtaposed electrospinning is not easy, especially when the electrostatic repulsive force between the two fluids is greater than the adhesive force, which is prone to nanofiber bifurcation [89]. Therefore, researchers need to carefully regulate the parameters of electrospinning or add external constraints to prevent the repulsive separation of the fluids after they are pushed out of the needle (Figure 5D) [88].

More surprisingly, on the basis of juxtaposed electrospinning, Xu et al. [90] prepared nanofiber mats with a three-layer structure by controlling the sequence and time of fluid feed injection, so that the nanofibrous mats with a three-layer structure were prepared by alternately single-fluid electrospinning and juxtaposed electrospinning. The alternately spun bifluids were PCL and gelatin mixed with nano-zinc oxide (n-ZnO) and loaded ciprofloxacin (CIP) drugs, respectively. The surface layer, hydrophobic polycaprolactone (PCL), possessed good hydrophobicity and resistance to external microbial adhesion. Hydrophilic Janus nanofibers prepared by side-by-side electrospinning in the middle layer. The bottom layer is made of hydrophilic gelatin, which provides a moist environment for wound healing. Biological wound healing exhibited accelerated collagen deposition, enhanced angiogenesis, and complete wound healing within 14 days. In conclusion, this combination of layered and juxtaposed spun structures has a strong potential to accelerate wound healing [91]. In addition, dual-fluid conjugate electrospinning facilitates the preparation of loose and porous fiber sponge structures [92].

Double-fluid electrospinning effectively enhances the mechanical strength and spinnability of electrospun fibers and achieves more effective control over the embedded drugs [93,94,95]. The structural diversity provides a good material foundation for the development of applications adapted to various fields. Bifluidically prepared nanofibers are used in applications such as piezoelectric catalysis, food packaging, filter membranes, battery separators, sensors, wound excipients, and carriers for catalysts or drugs [29,60,96,97].

#### 3.2.3. Multi-Fluid Electrospinning

**Figure 6:**
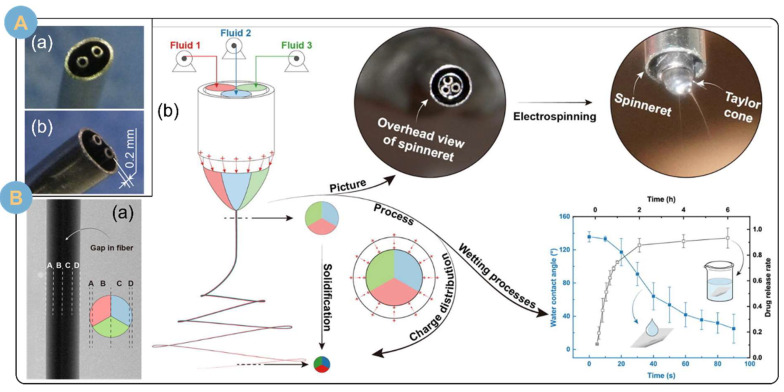
(**A**) Design of an electrospun spinneret with a “pig snout” three-fluid homogeneous shell-separated double core: (**a**) front view; (**b**) side view; structural outlets from three inlets [101]. (**B**) (**a**) Preparation of chimeric Janus microfibers with a specially designed multi-fluid complex spinneret and detection of their wettability; (**b**) TEM images of chimeric Janus microfiber [30].

The necessity of multifluid electrospinning research is gaining widespread attention in order to equip electrospun filaments with more diverse performances and to realize effective responses to complex scenarios. Multifluid electrospinning strategies with various structures have been developed, such as tertiary coaxial structures, pig-nose structures, and Janus structures with shells [29,93,98]. Multifluid electrospinning is an expansion of bifluid electrospinning, so multifluid electrospinning also possesses the properties of bifluid electrospinning, such as structural spinning aids and structural controlled release. In addition, multifluid electrospinning greatly enriches the variety and number of polymers spun, which is a popular direction for further research and development [99]. However, there are some difficulties in electrostatic spinning of nanofibers with tertiary structures, such as the difficulty of electrospinning and forming the tertiary structure. Observing and proving the tertiary structure is also a problem that needs to be solved. Facing the unobservability of the tertiary structure, Wang et al. [100] facilitated the visualization of the tertiary structure by using a laser to excite the stain. From inside to outside, the three fluids were glycerol mixed with doxorubicin (DOX), poly (ε-caprolactone) (PCL) mixed with tricaprylyl, and poly (d,l-propyl cross-linker) (PDLLA). The fibers were molded as glycerol ellipsoids encapsulated inside the fibers. In addition, glycerol was stained with rhodamine B, PCL solution was stained with fluorescein isothiocyanate (FITC), and PDLLA was stained with coumarin 120. Confocal laser scanning microscopy (CLSM) allowed the observation of the tertiary structure of the nanofibers by observing the distribution of fluorescence intensity displayed by the stains after excitation by 561 nm, 405 nm, and 488 nm lasers. Three-fluid electrospinning realized the simultaneous loading of hydrophilic drug (DOX-HCl) and hydrophobic drug (trichloronaphthamide). Moreover, the nanofibers prepared by electrospinning were sandwiched between two layers of gel sponges. The ingenious sandwich structure not only served as an excellent osmosis-absorbing and hemostatic property, but also synergized the slow and continuous release of the anticancer drugs DOX and trepanolactone to inhibit and kill residual cancer cells. In another experiment, Chang et al. [101] fabricated their own spinning head with a pig snout structure (Figure 6A) and prepared a novel three-cavity composite structure by using a three-fluid electrospinning technique. The composite structure had a common outer shell with two independently separated cores in the middle. The average diameter of the fibers prepared by electrospinning was 660 ± 160 nm, and the ideal structure was clearly observed under SEM and TEM, which provided a good platform for the realization of three-phase intelligent drug-controlled release. In addition, Wang et al. [30] prepared chimeric Janus microfibers (Figure 6B(a)) with an average diameter of 1.370 ± 0.280 μm by multifluid electrospinning using a specially designed composite spinneret (Figure 6B(b)). The PVP and CA were polymer matrices, and the ratio of which was adjusted to allow the fibers to form three parts with a wettability gradient, and the theory of the differences in wettability and water movement driven by the wettability gradient was used to explain the differences in the reduction of the water contact angle (WCA) of various fiber membranes. Given the wettability gradient, the rate of drug release was positively correlated with the rate of decrease in the WCA of the embedded Janus fibers. In addition, four fitting models were used to match the drug release profiles, indicating that the electrospun fibrous membranes were all slow-release systems. The embedded Janus fibers are amenable to two strategies for improving wettability, including the construction of wettability-differentiated surfaces and the addition of readily hydrophilic materials, and they are expected to be used in the fabrication of mist collection membranes and humidity sensors.

The electrospun fibers prepared by single-fluid, two-fluid, and multi-fluid electrospinning had diverse internal structures and morphologies, as shown in Table 1. The single-fluid electrospinning was easy to prepare, the fibers were uniform, and the drug could be added directly to the precursor. The fibers prepared by two-fluid monospinning have a core-sheath or Janus structure, which can improve the spinnability of natural polymers on the electrospun fiber structure. Moreover, different layers loaded with drugs can provide a good drug-carrying platform for the controlled release of drugs. Multi-compartmental nanofibers prepared by multifluid electrospinning make it possible for more diverse drug release strategies.

**Table 1:** Diameter and morphology of structurally diverse electrospun fibers.

Nanostructure	Polymer	Fluid	Loading	Diameter/nm	Morphology	Ref.
Single-spun fiber	PVS/SA	deionized water	GO, ZnO	199 ± 22	Smooth, unbranched, uniform fibers	[64]
	SA/PEO	deionized water	ZnO NPs	100 ± 30	Forked, subuniform, sporadic beaded fibers	[70]
	PVA/gelatine/SA	2-Propanol, DDW	collagen	229	Smooth non-beads uniform fiber	[74]
	CD	water, mellitic acid, SHP	graphitic acid linker	460 ± 49	Smooth sub-homogeneous fibers	[75]
Core-sheath construction	Core—CS Shell–SA/PVA	acetic acid	Asiaticoside	ID = 98.1 OD = 168.5	Smooth subuniform fibers with bifurcated core-shell structure	[84]
	Core—PVA Shell-SA/PEO	water	sCT	241 ± 38	Smooth, beadless fibers with uniform core-sheath structure	[85]
Janus structure	PVP//CA	Ethanol and acetic acid	KET//AgNPs	780 ± 180	Uniform and smooth Janus structural microfibers	[87]
Chimeric Janus structures	PVP CA	acetone/ alcohol/DMAc	KET	1370 ± 280	Smooth, beadless, uniformly embedded Janus microfibers	[30]
“Pig-nose” structure	Shell-E100 (EE) Core 1-EL Core 2-ES	anhydrous ethanol/ DMAc	PAR	660 ± 160	Smooth and homogeneous fibers with a shell-separated double-core structure	[101]

## 4. Electrospun Nanofibers for Drug Delivery and Release

Electrospun nanofibers are popular in the field of drug delivery due to their diversity of materials, structures, and high porosity after deposition into a film [102]. According to the different judgment perspectives of drug release, such as drug release rate, release duration, and delivery release location, it can be divided into different drug delivery release modes [103]. This review categorizes drug delivery release modes into pulsatile release, sustained release, biphasic release, stimulus-responsive release, targeted release, and other release modes. Pulsed release is also rapid release or burst release, where the loaded drug is rapidly released from the electrospun nanofibers to reach the effective zone of the drug. Sustained release is the slow sustained release of the loaded drug to reduce the number of drug uptakes and maintain the drug concentration in the internal environment and therapeutic effect. Slow release is a design and development direction of wide interest to researchers [103] and plays a role in many fields, such as regenerative engineering, tissue engineering, food engineering, and agricultural fertilization [104,105,106,107]. Biphasic release is a staged combination of pulsed and slow release, which can quickly reach the minimum effective concentration of the drug while avoiding drug toxicity caused by sustained rapid release. Electrospun nanofibers with stimuli-responsive release properties will intelligently change the release rate of loaded drugs according to changes in the pH of the external environment [108,109]. Stimulus-responsive release undoubtedly reduces the amount of drug, improves the accuracy of the location of drug delivery and release, and avoids erosion of the drug by the adverse environment. Targeted release is mainly aimed at the delivery of targeted drugs, targeted delivery to the tumor location, inhibiting tumor cell regeneration, or killing tumor cells [110].

**Figure 7:**
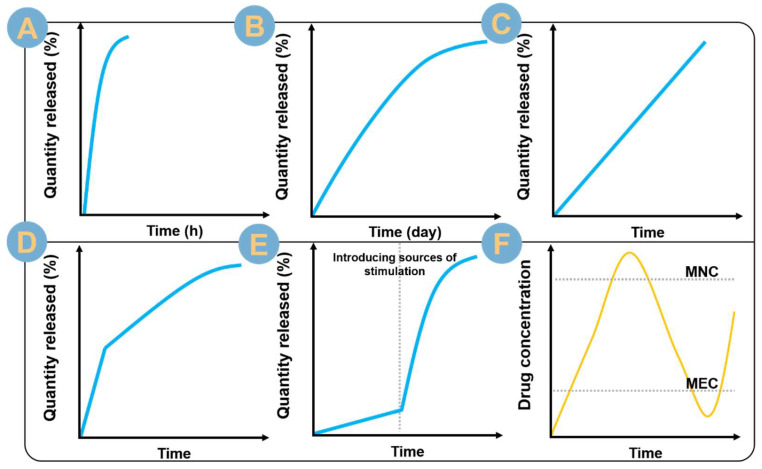
(**A**–**E**) Schematic of five trends in drug release: (**A**) Pulse release; (**B**) Sustained release; (**C**) zero-order release; (**D**) biphasic release; (**E**) Response Release; (**F**) Effective therapeutic range of drugs, Minimum Effective Concentration (MEC), Maximum non-toxic concentration (MNC).

There are five general trends in the in vitro release profiles of drugs (Figure 7): line A: pulsatile release; line B: conventional slow release; line C: zero-order release (due to zero-order kinetics); line D: biphasic release; and line E: stimulus-responsive release. The release profile reflects the control of the drug by the drug delivery system, but more importantly, the therapeutic effect of the drug, and the attainment and stabilization of the in vivo or local drug concentration within the therapeutic window (Figure 7F). The process of releasing a drug that has not reached the minimum effective concentration (MEC) wastes the drug, so every effort is made to shorten the duration of this process. The drug release process that exceeds the maximum non-toxic concentration (MNC) is a breakthrough to the safe concentration of the drug, so every effort is made to circumvent this process. In conclusion, designing a suitable drug delivery system to achieve accurate, efficient, non-toxic, and convenient drug delivery release is a goal that researchers are constantly pursuing.

## 5. Electrospun Alginate Nanofiber Delivery and Release Modes

After the previous sections, it is easy to see that SA has good hydrophilicity and gel-forming ability, as well as high biocompatibility, non-toxicity, and biodegradability. The electrospun nanofibers are easy to prepare and have a variety of structures, and the fiber-converted nanofibrous membranes have a high specific surface area and can be adjusted to obtain a suitable void ratio by adjusting the electrodischarge parameters. With the strong combination of the two, electrospun alginate nanofibers (EANFs) undoubtedly have great potential for application in many fields. However, the non-spinnability of pure SA is in urgent need of a solution strategy. After continuous exploration by researchers, other polymers (PEO, PVA) were used as co-spinning agents, and surfactants were used as auxiliary spinning agents to improve the spinnability of SA. On the other hand, SA is characterized by inter- and intramolecular hydrogen bonding [21], rigid chain conformation, and low solubility. Researchers need to modify the alginate backbone, such as oxidation, sulfation, and esterification of the hydroxyl group, by replacing the proton of the hydroxyl group with a new atom or functional group that lacks the ability to form hydrogen bonds. Alginate backbone modifications likewise improve the spinnability of SA solutions. For example, Daemi et al. [40] synthesized sulfated sodium alginate (SSA) by sulfating the hydroxyl functional group of SA. SSA reduces the hydrogen bonding density and improves spinnability relative to SA. In addition, SA electrospinning into filaments was likewise promoted by the aid of the diverse structures of electrospun nanofibers [4]. Post-processing methods, such as the template method, can also be used to use nanofiber membranes as substrates for SA surface binding, ultimately forming a composite SA nanofiber drug delivery system. EADDS were constructed to make it possible for drugs to achieve pulsatile release, sustained release, biphasic release, stimulus-responsive release, targeted release, and other release modes. Electrospun alginate nanofiber-loaded drugs include antibiotics [111], non-steroidal drugs [112], plant extracts [113], vitamins [114], H_2_S donors [19], and biological proteins [115].

### 5.1. Pulse Release

Pulse release is a specific drug delivery strategy that utilizes the release of a high concentration of a drug over a short period of time to achieve a therapeutic effect. Acute injuries and sudden blood spills require rapid drug delivery for effective infection control. Kitaria et al. [111] prepared composite nanofiber transdermal patches using electrospinning of a blend of polyvinyl alcohol (PVA) and sodium alginate (SA). PVA/SA nanofibers were loaded with the small-molecule antibiotic drug, ciprofloxacin, to achieve rapid drug release. In vitro drug release experiments showed that the patch followed the drug release model of Higuchi and Korsmeyer-Peppas. In vivo experiments showed hydroxyproline production at the wound site, promoting wound healing five days earlier. In a subsequent study, Najafi et al. [113] selected the same polymer electrospun for loading cardamom extract (CE). The average diameter of the drug-loaded nanofibers was 233 ± 33 nm. CE showed pulsatile release in the PVA/SA nanofiber drug delivery system with a rapid release of 86% of the drug within 24 h, and the release followed first-order kinetics. The pulsed release of the drug increased the antibacterial activity by about 99% against Gram-positive and 97% against Gram-negative bacteria, which resulted in rapid control of wound infections and good biocompatibility to promote wound healing.

**Figure 8:**
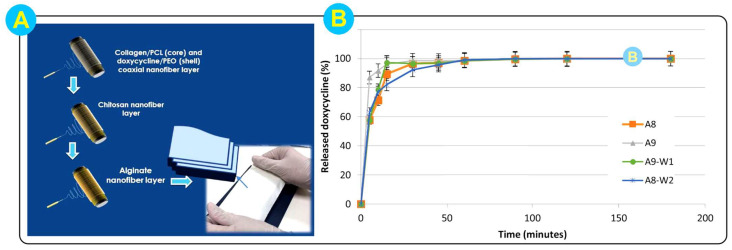
(**A**) Schematic of the electrodischarge nanofiber triple-layer wound dressing; (**B**) Release profiles of electrospun fiber-loaded doxycycline from coaxial electrospun fibers and three-layer wound dressing. A8:1.5% PCL + 4.5 % Collagen; A9:1% PCL + 4.5% Collagen; A9-W1: A9 coaxial nanofibers + SANFs + CS nanofibers; A8-W2: A8 coaxial nanofibers +  SANFs + CS nanofibers [116].

The special electrospun structure provides an excellent strategy for the controlled release of drugs. Zhu et al. [84] successfully prepared electrospun nanofibers with a shell and core structure by using a coaxial electrospinning process. A blend of alginate and polyvinyl alcohol (PVA) was used as the shell layer, and a CS solution loaded with Centella asiatica glycosides was used as the core layer. The shell- and core-structured nanofibers loaded with asiaticoside showed a faster drug release rate dominated by Fick diffusion as compared to the triterpenoid asiaticoside paste. The drug release from the shell-core structured nanofibers was also greater, with 20% more drug released within 12 h. Biological experiments showed that drug-loaded coaxial nanofibers significantly promoted the healing effect in rats with deep burns. The release of asiaticoside promoted the positive expression of vascular endothelial growth factor (VEGF), cluster of differentiation 31 (CD31), and proliferating cell nuclear antigen (PCNA), while down-regulating the expression of tumor necrosis factor (TNF) and interleukin-6 (IL-6). In addition to the design of the fiber structure, the combination of technologies can also improve the loading drug and pulse release capacity. Parın et al. [114] electrosprayed folic acid (FA) nanoparticles on poly (vinyl alcohol)-alginate (PVA-Alg) electrospun fibers by a strategy combining electrospinning and electrospraying technologies. More than half of the FA was released from the composite delivery system within 8 h in an artificial sweat (pH 5.44) environment. Cytotoxicity tests showed that the composite fiber-particle system exhibited good biocompatibility. The composite drug delivery system enables the pulsatile release of vitamins and opens up the application of skin care patches. The multilayer nanofiber membrane drug delivery system was designed to achieve the same effect of pulsatile release of drugs. Tort et al. [116] prepared a three-layer nanofiber wound dressing. The three-layer composite nanofiber membrane drug delivery system was flanked by electrospun fiber membranes with a core-shell structure and electrospun alginate fiber membranes, respectively (Figure 8A). Sandwiched between the two layers was a chitosan electrospun fiber membrane. The core-shell structure of the nanofiber shell layer was loaded with 2.5% doxycycline (DOX). The content of DOX in the three-layer composite nanofiber membrane was 260 μg/cm^2^. The in vitro release results showed that the drug was completely released within 15 min (Figure 8A). The nanofiber membrane showed no cytotoxic effect on the cells of the keratinocyte line. Moreover, the morphology, mechanical, bioadhesive, and wettability properties of the three-layer composite nanofiber membranes also maintained good stability, which has great potential for the application of wound dressings.

With the development of society and civilization, there is an increasing concern for mental and emotional health [117,118]. The dicyclic atypical antidepressant drug venlafaxine was released via an EADDS. Mann et al. [119] regulated and optimized the concentrations of SA, sodium carboxymethylcellulose, and poly(vinyl alcohol), and electrospun prepared oral mucosa with easy adhesion. The drug was uniformly distributed on the surface of the electrospun fiber. The fibers had high mechanical strength, weak acidic surface, and good biocompatibility. The results of the drug release test showed that the maximum release rate of venlafaxine at 5 h was 89.97%. The drug release mechanism of the electrospun drug-carrying mucosa in the rabbit oral cavity was consistent with the pure Fick’s diffusion model, and the release was 10% higher relative to that of the solvent-cast patch.

EADDSs enable the pulsatile release of drugs. The ECM-like property of SA improves the biocompatibility of EADDS. Pulse release releases a large amount of drug in a short period of time, which is conducive to the rapid arrival of the drug concentration at the minimum effective concentration (MEC), and provides a rapid-acting therapeutic effect. To achieve rapid drug release, single fluid electrospinning is generally used. When electrospinning a core-shell structured fiber drug delivery system, the drug is loaded into the shell layer to facilitate the contact and diffusion of the drug with the external environment. In addition, in composite multilayer electrospun fiber membranes, the drug is also placed in the outer layer. However, the high rate of drug release in a short period of time can bring non-safe therapeutic effects, such as toxic side effects, to living organisms if the maximum non-toxic concentration (MNC) is breached. Therefore, effective control of drug release needs to be taken care of to avoid toxic side effects. Novel controlled release delivery systems need to be designed to ensure therapeutic efficacy and safety.

### 5.2. Sustained Release

Sustained release is a sustained and slow drug release, which can also be referred to as sustained drug release (SDR) in order to achieve safe, effective, long-lasting, and convenient drug delivery effects. The development of a complex structure electrospun nanofiber process to produce multi-chambered fibers in one step has received widespread attention as a new strategy for the development of novel SDR nanomaterial platforms [103,120]. EANFs are ideal drug delivery carriers for sustained drug release [26,27] thanks to the stability of SA and the controllability of electrospun copolymers. The electrospun alginate nanofiber drug system allows for prolonged and even sustained drug release for up to days or even months.

Drugs can be doped in SA electrospun precursor solutions to achieve a slow-release effect. For example, nonsteroidal anti-inflammatory drugs can achieve a good slow-release effect by a SANF system. Chen et al. [112] prepared alginate derivatives (RAOA) by modification through the redox-amidation reaction, and then the composite was prepared by electrospinning with poly(vinyl alcohol) (PVA) as the co-spun polymer. nanofibers. Ibuprofen was loaded into the RAOA/ PVA electrospun nanofibers. The oxidative-reducing amination reaction not only breaks the intramolecular hydrogen bonds of SA but also increases the affinity for hydrophobic ibuprofen. The rate of drug release is affected by the difference in the volume ratio of RAOA/PVA. Rapid release of ibuprofen in unmodified SA/PVA (50/50) electrospun fibers in pH 7.4 PBS medium at 37 °C released approximately 90% of the drug in 150 min. Modification and adjustment of the polymer ratio resulted in a sustained and substantial increase in the drug release time to 810 min due to the enhanced solubility and encapsulation of ibuprofen by the polymer. In addition, RAOA/PVA electrospun composite nanofibers exhibited low cytotoxicity to L929 cells. The slow-release EADDS enables the stable maintenance of antibiotic-like drug concentration at the wound site for a longer period of time, which improves drug utilization and sustainably promotes wound healing. Penton et al. [121] successfully loaded the antibiotic ciprofloxacin with SA/PVA electrospun nanofibers to achieve sustained release for up to 14 days.

**Figure 9:**
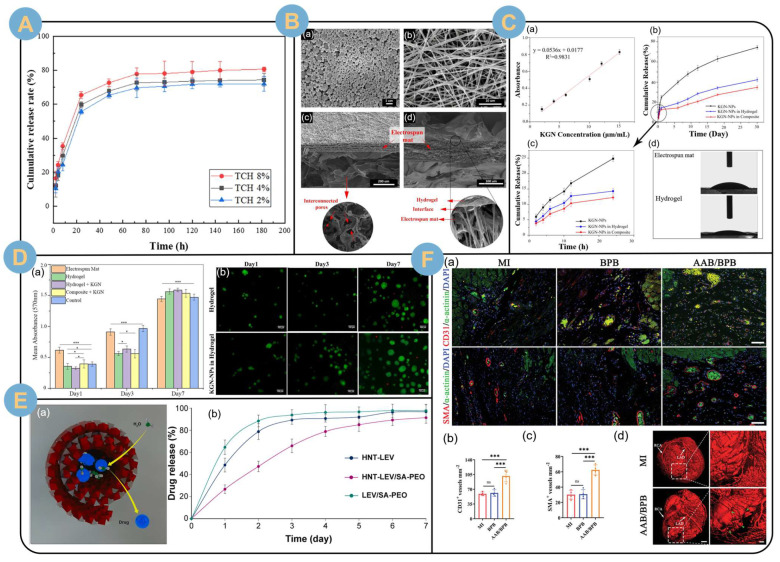
(**A**) Release rate of composite nanofibers loaded with different TCH contents [117]. (**B**) FE-SEM images. Transverse images of (**a**) drug-loaded nanoparticles, (**b**) PCL electrospun fibers, (**c**) composite fiber mats, and (**d**) multilayered composite scaffolds (electrospun mats are indicated by arrows) [118]. (**C**) (**a**) KGN calibration curves, (**b**) cumulative release profiles of KGN from particles, hydrogels, and composites over 30 days, (**c**) cumulative release profiles of KGN from particles, hydrogels, and composites over 24 h, and (**d**) images of the water contact angle of electrospun mats and hydrogels [118]. (**D**) (**a**) Raisinomycin assay of scaffolds with different culture times (Values represent the mean ± SD, n = 3, * *p* < 0.05, *** *p* < 0.001). (**b**) Live/dead cell viability assay after 1, 3, and 7 days on Alg:Alg-Sul hydrogels with or without KGN-NPs [118]. (**E**) (**a**) Schematic of the release mechanism of LEV from nanohybrids. (**b**) Drug release profiles from different fractions of fibers over 7 days [65]. (**F**) (**a**–**d**) Sections of the infarct border zone on day 28 of culture were collected and immunofluorescently stained for the endothelial marker CD31 and α-smooth muscle actin (SMA) (*** *p* < 0.001; ns, nonsignificant). (**a**) Cardiomyocytes were stained with SMA, and nuclei were counterstained with DAPI. Scale bar: 75 μm. Border zone vessel density (**b**) was quantified as the expression of CD31; arteriolar density (**c**) was quantified as the number of vasculature-like structures expressing SMA (n = 4). (**d**) Images of micro-CT scans after imaging with Microfil. Scale bars: 2 mm and 500 µm [19].

The structural design contributed to the slow release of the drug [122]. Huang et al. [117,118,119,120,121,122,123,124,125] electrospun poly(L-propylcaprolactone CO)-ε-caprolactone) (PLCL) nanofiber membranes loaded with tetracycline hydrochloride (TCH). The TCH-loaded PLCL fiber membrane was then used as an intermediate layer sandwiched between two layers of polyethylene oxide (PEO)/sodium alginate (SA) electrospun nanofiber membranes to form a three-layer composite drug-carrying wound dressing. The multilayer composite drug-carrying wound dressing was able to provide good control of wound exudate while achieving a sustained release of the drug for more than 10 days (Figure 9A). The sustained, slow release of the drug inhibits bacterial reproduction while preventing wound infection, which can be used for the treatment of chronic skin diseases and wound healing. The coaxial structure electrostatic spinning technique also played an excellent role in slow release of drugs. Rezaei et al. [4] prepared single electrospun and core-shell structured nanofibers loaded with vitamin C (VC) using PEO/SA as precursor solution. Drug release experiments showed that the core-shell structured nanofibers displayed a better retardation effect on the release of VC than the hybrid single electrospun nanofibers. The core-shell structured EADDS slowed down the penetration rate of VC and provided an excellent carrier platform for drug delivery for the treatment of pigmented purpuric dermatosis (PPD). Janus-structured bilayered nanofiber membranes could achieve a stable and slow release of siRNAs targeting FK506-binding protein-like (FKBPL). Mulholland et al. [123] bilayered electrospun fibrous wound patches using polyvinyl alcohol mixed with CS and SA as precursors, respectively. siFKBPL was condensed with the novel cationic 30-mer amphipathic peptide RALA to form nanoparticles that were loaded in the electrospun fibers. The sustained and stable slow release of the nanoparticles (48 h) significantly promoted the growth of blood vessels in mouse wounds, with a 326% increase in vessel density.

Different technology combinations create more possibilities for electrospun SA sustained-release drug delivery systems. Penton et al. [121] electrosprayed ciprofloxacin-loaded oxidized alginate/gelatin particles on polyaniline (PANi)/polycaprolactone (PCL) electrospun fiber membranes. The fiber/particle combination drug delivery system achieved sustained release of ciprofloxacin with a good antimicrobial effect. Capsaicin, which has an inhibitory effect on tumor cell proliferation, can likewise be prolonged for release by the fiber/particle combination drug delivery system. Ahmady et al. [124] first prepared SA nanoparticles loaded with capsaicin (Cap) using cationic bicosubsurfactants and nanoemulsion templates. The average diameter of the drug-loaded nanoparticles was about 19.42 nm, and the encapsulation rate was as high as about 98.7%. The drug-loaded nanoparticles were hybrid electrospuns in polycaprolactone-chitosan nanofibers. The release time of Cap loaded in the fiber/particle combination drug delivery system was prolonged by 4.1-fold, achieving a sustained release of more than 500 h (21 days). Biological assay results demonstrated that the Cap-loaded fiber/particle combination drug delivery system effectively inhibited the proliferation of MCF-7 human breast cells while showing excellent cytocompatibility with human dermal fibroblasts (HDF). The slow-release drug delivery system for Cap has great potential for long-term cancer prevention and treatment. The drug delivery system combining electrospun fiber membrane and SA gel sponge can significantly reduce the initial burst release of the drug and promote prolonged sustained slow release. Zare et al. [118] designed electrospun fiber-alginate sulfate gel scaffolds to load Kartogenin-PLGA (poly(lactic-co-glycolic acid)) nanoparticles (KGN-NPs) (Figure 9B). The composite drug delivery scaffolds possessed good mechanical properties and hydrophilicity (Figure 9C(d)). Thanks to the novel structure of the sponge-clamped nanofiber membrane being designed, the initial burst release of KGN was reduced by 8% and sustained slow release was achieved for more than 30 days (Figure 9C(b)). In addition, the reinforced composite scaffold possessed good degradability, as well as good biocompatibility (Figure 9D). The composite scaffold drug slow-release system is expected to advance the field of cartilage and tendon bone regenerative healing.

Drugs can be electrospun not only by being directly mixed in SA precursor solution, but also by being loaded on the nanotubes first and then mixed into the precursor solution. Fatahi et al. [65] mixed levofloxacin (LEV)-loaded halloysite nanotubes (HNTs) in sodium alginate (SA)-poly ethylene oxide (PEO) precursor solution mixed electrospun nanofibers (Figure 9E(a)). The in vitro release profile showed that LEV was slowly released from the HNT-LEV/SA-PEO nanocomposite fibers. The release time was prolonged up to 7 days compared to the electrospun LEV/SA-PEO nanofibers with the drug unloaded on the nanotubes (Figure 9E(b)).

In addition to direct drug delivery, researchers are exploring slow-release delivery systems for gaseous signaling molecules. Li et al. [19] integrated SA modified with 2-aminopyridine-5-thiocarboxamide (H_2_S donor) into an albumin electrospun fiber complex scaffold. H_2_S was slowly released from the complex scaffold to protect the myocardium by maintaining the concentration of hydrogen sulfide for at least 21 days. H_2_S was slowly released from the complex scaffold to protect the myocardium by maintaining a higher concentration of hydrogen sulfide for at least 21 days. This study also demonstrated that a complex scaffold enhanced the regenerative capacity of M2 macrophages and attenuated the inflammatory polarization of macrophages by reducing intracellular reactive oxygen species (ROS). vascular and arteriolar densities in the border region of complex scaffold/BPB (black phosphorus nanosheet-loaded albumin scaffolds)-treated hearts remained significantly higher than that of BPB-treated or nontreated hearts (Figure 9F), which contributes to the establishment of collateral circulation (as shown by the green triangles are shown) (Figure 9F(d)). The engineered cardiac patch enabled the composite electrospun SA stent to achieve effective release of gaseous drugs, alleviate the inflammatory response after myocardial infarction, and promote angiogenesis. The design of composite stents provides a promising therapeutic strategy for restoring cardiac function and treating myocardial infarction. In another study, heparin was loaded onto SA electrospun nanofiber scaffolds by covalently modified post-treatment to achieve sustained and effective slow release for one week [125]. Degradable nanofiber scaffolds for tissue engineering maintain the sustained release of heparin-binding growth factors through affinity interactions.

### 5.3. Biphasic Release

Biphasic release is an ingenious method of drug delivery. The sustained release of large quantities of a drug with high blood levels can cause toxic side effects, while insufficient blood levels do not achieve a therapeutic effect [126,127]. Researchers have cleverly designed drug-carrying platforms for biphasic release. The first phase is usually a pulsatile release to rapidly reach the minimum effective concentration (MEC) of the drug; the second phase is a slow release, which avoids the drug concentration exceeding the maximum non-toxic concentration (MNC) caused by the rapid release of the drug for too long. At the same time, the sustained slow release of the second phase maintains the stability of the drug concentration in the environment and provides a continuous therapeutic effect. The biphasic release approach improves the bioavailability of the drug while reducing the number of doses and the toxic side effects of the drug.

Biphasic release can be achieved by the phased release of a single drug. Electrospun nanofibers loaded with plant extract drugs are gradually being emphasized [128], and Shakibania et al. [129] prepared a composite drug delivery system by coating PVA/SA on electrospun curcumin-loaded poly(lactic acid) (PLA) nanofiber membranes. The hydrophilic coating reduced the burst release of curcumin and prolonged the overall release time (slow release after about 270 min), realizing the effect of biphasic release. Anti-cancer drugs can similarly be delivered by EADDS to achieve a biphasic release, with a pulsatile release in the first 10 h and a slow release immediately followed by 134 h [130]. Glycemic control is an important challenge in the life of diabetic patients. Sharma et al. [131] prepared polyvinyl alcohol (PVA)/sodium alginate (NaAlg) sublingual transmucosal patch loaded with the antidiabetic drug insulin using electrospinning technique. Insulin was initially released in very slight bursts, followed by a sustained slow release for about 10 h. The release followed first-order kinetics. Biological tests validated the effect of patch administration by the sublingual route in male Wistar rats, and the release remained consistent with in vitro release, with effective control of blood glucose concentrations. The electrospun SA nanofiber sublingual patch provides a new delivery platform for non-orally available insulin. Psychotropic drugs can also be delivered by the EADDS to achieve the effect of biphasic release [119].

**Figure 10:**
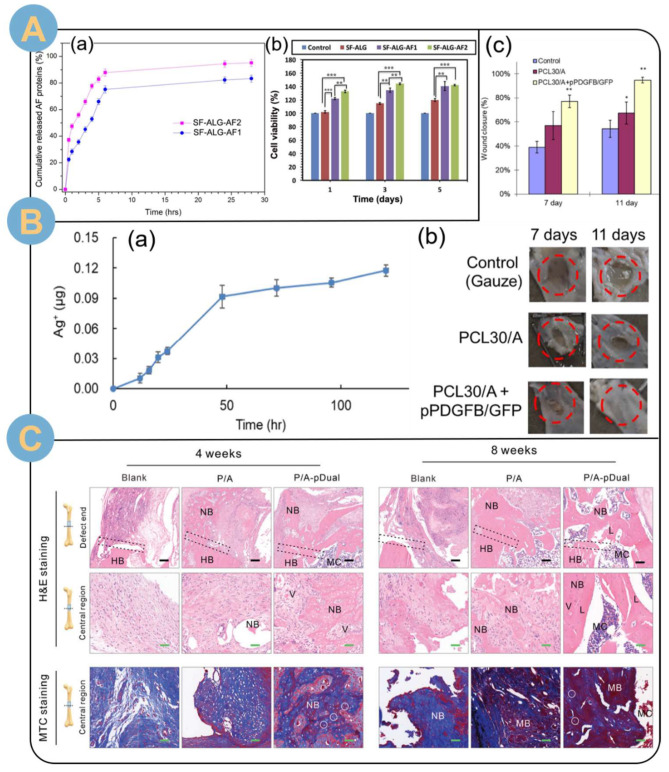
(**A**) (**a**) Release profile of AF protein from composite electrospun fiber dressing. (**b**) MTT assay of different composite dressings during 5-day incubation (** *p* < 0.01, *** *p* < 0.001) [115]. (**B**) (**a**) The quantification results of released silver from PCL30/A in PBS at 37 °C. (**b**,**c**) In vivo study of wound healing using composite fibers. (**b**) The healing of wounds covered by different dressings. Red dashed circles indicate the size of the wound created. (**c**) The quantification results of the wound closure (* *p* < 0.05. ** *p* < 0.01 compared to the control group) [20]. (**C**) Histologic analysis of bone repair: H&E staining and MTC staining at 4 and 8 weeks after implantation. White circles: osteoblasts; white arrows: Haver’s canal; HB, NB, and MB are host, new, and mature bone, respectively; MC is bone marrow cavity; L is lamellar; and V is vascular. The black scale bar is 200 μm, and the green scale bar is 50 μm [135].

Designing fiber-gel composite drug delivery systems is an ingenious strategy for drugs to achieve biphasic release and maintain the biological activity of drugs [132]. Protein-based drugs can be released biphasically by an electrospun fiber-SA gel drug delivery system. Ghalei et al. [115] covered alginate hydrogel (ALG) on filipin (SF) electrospun fibers loaded with amniotic fluid (AF). The EADDS was in the form of a tightly fused fibrous dressing after cross-linking and freeze drying. The in vitro release results demonstrated a biphasic release of AF, with an initial phase of pulsatile release of AF followed by a slowing down of the release rate (Figure 10A(a)). The electrospun SA composite drug delivery wound dressing with biphasic release of AF demonstrated safety and non-toxicity in cellular assays (Figure 10A(b)) and contributed to fibroblast adhesion, spreading, and proliferation. In another study, Nematpour et al. [133] prepared nanofibrous vaginal patches loaded with clotrimazole (CLZ) using two-fluid electrospinning with dextrose (Dex) and SA/PVA electrospinning. Compared with CLZ-loaded films, the EADDS was flexible and allosteric, and plasticizers with cytotoxic side effects were not required. Approximately 30% of the CLZ in the EADDS is released to the external environment in an initial burst within 5 min. Up to a total of 98% of the clotrimazole was sustained for 30 min, and the release profile followed a zero-order model of uniform release. The concentration range of CLZ inhibited the growth of *Candida albicans* (ATCC 76615) and *Candida dubliniensis* (DSM 13268) within 125–250 μg/mL, and killed them at 125–500 μg/mL (*p* ≤ 0.01).

In a new study, coding DNA can be efficiently delivered and expressed by EADDS, for example, coding for platelet-derived growth factor-B (PDGF-B). Hu et al. [20] electrospun prepared SA/PCL composite nanofibers. Meanwhile, nanosilver was loaded in the PCL polymer fraction, and plasmid DNA nanocomplexes of cationic polyethyleneimine (PEI) and PDGF-B were adsorbed on the anionic alginate fraction. Immersed in PBS, the nanosilver showed two stages of being released (Figure 10B(a)): a rapid release in the first 24 h and a slow release in the immediately following 120 h. The nanosilver was released in the first 24 h, and the nanosilver was released in the second 24 h. The composite drug delivery system facilitated the internalization of adherent cells for in situ PDGF-B transfection. PDGF-B expressed by transfected cells significantly promoted cell proliferation. Biological experiments showed that the cross-linked SA/PCL composite drug-loaded wound dressing could effectively promote wound healing (Figure 10B(b,c)), whereas PDGF-B expressed by in situ transfection could significantly increase collagen formation, thereby promoting tissue regeneration.

EADDSs can be loaded with bi-drugs to achieve biphasic drug delivery. Loading dual drugs with different hydrophilic and hydrophobic properties to achieve biphasic release is also an excellent strategy [133,134,135]. Chen et al. prepared gelatin/oxidized alginate nanofibrous membranes by a combination of electrospinning and cross-linking techniques, and the nanofibrous membranes were simultaneously loaded with both hydrophilic Gentamicin Sulfate (GS) and hydrophobic ciprofloxacin drugs. The dual-loaded gelatin/oxidized alginate drug delivery system could achieve complete release of GS within 6 days and sustained release of ciprofloxacin for more than 3 weeks. The simultaneous loading of dual drugs was effective in providing antimicrobial effects and promoting wound remodeling regeneration and healing within 21 days compared to fibers loaded with a single drug. The core-shell structure of electrospun fibers facilitates biphasic drug release [134]. The flow rate of the sheath determines the amount of loaded drug released at different stages of biphasic release. In addition to the simultaneous loading of hydrophilic and hydrophobic drugs, the EADDS can achieve effective loading and biphasic release of bi-genes. He et al. [135] electrospun composite fiber scaffolds with poly(ε-caprolactone) sheaths and SA hydrogel cores. The core layer of SA hydrogel encapsulated PP@KALA nanocomplexes carrying pVEGF (PP@KALA-pVEGF). pVEGF was released by rapid diffusion at 3 days due to rapid diffusion of DNA molecules and breakdown of the SA hydrogel. The pCL fiber sheaths were encapsulated with polydopamine (PDA) and loaded with the pBMP2 gene. The pBMP2 was released at a sustained slow rate over a period of 21 days. Efficient delivery and biphasic release of both genes promoted in vitro osteogenesis, as evidenced by increased calcium deposition and osteogenic gene expression, with a significant increase in calcified bone tissue (red) and gradual maturation at 8 weeks (Figure 10C).

### 5.4. Response Release

Smart-responsive drug delivery systems enable drug release under specific conditions, reduce drug loss in non-acting environments, and make drug delivery more precise [136,137]. Thanks to the sensitivity and stability of SA to pH, EADDSs with stimuli-responsive properties have been gradually explored for development [27].

**Figure 11:**
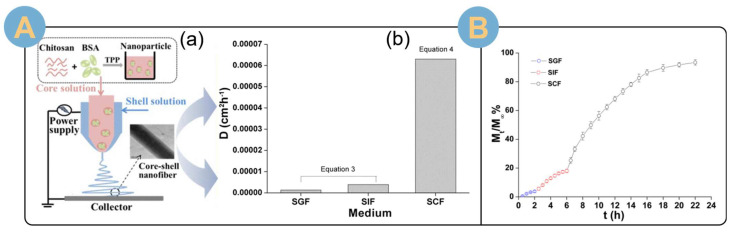
(**A**) (**a**) Schematic diagram of the preparation of electrospun nanofibers with a core-shell structure, using SA as the shell layer and previously prepared CS nanoparticles loaded with BSA as the core layer. (**b**) Diffusion release coefficients of BSA in SGF, SIF, and SCF media [108]. (**B**) Simulation of BSA release from electrospun fibers during transport from the oral cavity to the colon [139].

The EADDS mainly places SA in the outer layer in order to achieve the effect of intelligent response release. Schoeller et al. [138] deposited a CS/SA coating on electrospun PLGA nanofibers to achieve pH-responsive release of the loaded drug ibuprofen. The in vitro release results showed that the response release drug delivery system loaded with ibuprofen released slower at acidic pH (1.0) than at neutral pH and could be loaded with the drug to bypass the acidic environment of the digestive tract to reach the colonic site for release. Also serving as a drug delivery platform for colonic drug delivery, Wen et al. [108] prepared electrospun nanofibers with a core-shell structure using SA as the shell layer and CS nanoparticles loaded with bovine serum albumin (BSA) as the core layer (Figure 11A(a)). In vitro simulated oral-to-colonic release profiles showed (Figure 11A(b)) that a small fraction of BSA (<4% for 2 h) was released in simulated gastric fluid (pH 1.2), 15% of BSA was released in simulated intestinal fluid (pH 6.8) for more than 4 h, and about 75% of BSA was released in simulated colonic fluid (pH 7.4) for 16 h. Core-shell-structured EANFs for drug delivery systems. The loaded BSA was released in response to simulated colonic fluid, demonstrating the stimulus-responsive drug release capability of the drug delivery system. The protein conformation indicated that the core–shell structure of the coaxial electrospun fibers protected the integrity of the BSA. In another study [139], the same coaxial EADDS improved protein bioavailability. Simulating the transit from the oral cavity to the colon (Figure 11B), most of the BSA could be released by erosion in simulated colonic fluid.

Stimulus-responsive release to acidic environments can be achieved by modulating Schiff base bonding and electrostatic adsorption. In a new study, Wang et al. [15] prepared stimuli-responsive electrospun fibers loaded with curcumin by alternating deposition of chitosan-dialdehyde-β-cyclodextrin-curcumin (CS-DCD-CUR) and alginic acid dialdehyde (ADA) on electrospun cellulose acetate (CA) nanofibers for a drug delivery system. The combined effect of Schiff base-bonded hydrolysis and reduced electrostatic attraction in an acidic ambient solution (pH 4.2) resulted in the cumulative release of curcumin up to 78.13%, which was much higher than that in a pH 7.4 ambient solution (22.0%).

The EADDS realizes stimulus-responsive release and avoids the erosion of drugs by non-releasing environments, which provides an excellent delivery platform basis for precise drug delivery and convenient transoral drug administration.

### 5.5. Targeted Release

The development of nanotechnology has led to the research and development of drug delivery carriers, and carrier platforms for targeted delivery have been designed one after another to minimize the harm of cancer to human health [8,110,140,141]. The EADDS achieves targeted drug delivery, which improves drug utilization and effectively circumvents the toxic side effects of systemic drug delivery.

Targeted EADDSs can deliver indication drugs to specified locations in the body via stimulus-responsive properties. For example, Wen et al. [142] electrospun prepared core-shell structured nanofibers with a shell layer of SA. Quercetin, an antioxidant that inhibits colon cancer, and prebiotics (oligogalactose), which promote intestinal stability, were loaded in the core layer of the fibers. EADDS can target drug delivery to the colonic location by oral administration. In another study, Feng et al. [85] coaxial electrospinning prepared core-sheath-structured electrospun fibers with SA in the shell layer for delivery of pectin-coated salmon calcitonin (sCT). Compared with single-spinning delivery of sCT, the coaxial structure was more resistant to erosion by simulated gastric fluid and would retain 88% of the drug bioactivity. In vitro release assays demonstrated sustained release of sCT from the pectin layer, with potential for colonic targeting.

**Figure 12:**
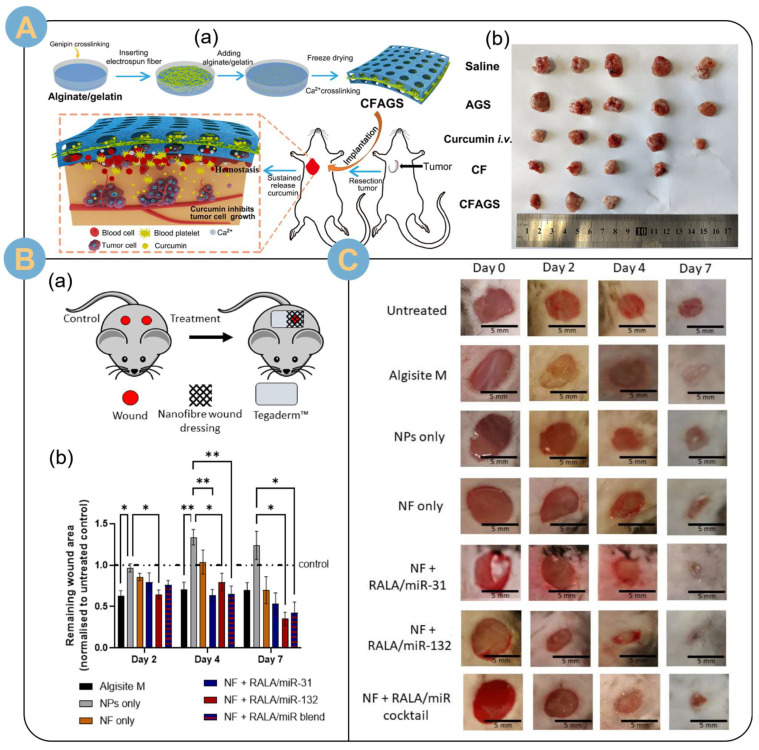
(**A**) (**a**) Schematic diagram of curcumin-loaded sponge-electrospun fiber-sponge sandwich structure for rapid hemostasis dressing and the prevention of tumor recurrence after surgery. (**b**) Dimensions of recurrent solid tumors on postoperative day 16 in different post-treatment groups [12]. (**B**) Two strategies for treating wound models. RALA/miR nanoparticles (NPs) containing 5 µg of miR and ALG/PVA/CIP nanofibers (NFs) containing 2.5 µg of miR. (**a**) Creation of a full-thickness wound model and comparison of treatments. (**b**) Remaining wound area at postoperative days 2, 4, and 7 in different groups after wound treatment. Data are reported as mean ± SEM, n = 5, and statistical significance is calculated by means of two-way ANOVA (* *p* < 0.05, ** *p* < 0.01). (**C**) Promotion of wound healing using different post-treatments. C57BL/6N mice to construct a thick incision wound-healing model. Representative images of wound closure on days 2, 4, and 7 were recorded. n = 5 [143].

The electrospun alginate targeted drug delivery system can also deliver the targeted drug by electrospinning fibers and alginate external appendage. Wei et al. [110] electrospun nanofibers loaded with the targeted drug, hydroxycamptothecin (HCPT), were lyophilized and chopped, and dispersed homogeneously in 2% SA solution for injection and drug delivery. The targeted drug also achieved a biphasic release effect with an initial release of about 20% and targeted sustained release for more than three weeks. Experiments in mice showed that fragmented fibers with a fiber length of 20 μm fragment HCPT had the best spatial distribution within the tumor, significantly promoting survival animals, inhibiting tumor growth, and inducing apoptosis of tumor cells. To promote rapid wound healing, the targeted drug curcumin was targeted and released to the wound site by an electrospun alginate targeted drug delivery system. Chen et al. [12] sandwiched curcumin-loaded nanofiber membranes between SA/gelatin sponges, and then freeze-dried them. A multilayer composite novel hemostatic sponge was prepared by electrostatic spinning and an interpenetrating polymer network (IPN) strategy (Figure 12A(a)). Coagulation and hemostasis experiments showed that the “Sandwich” drug delivery system was more effective than commercially available gelatin hemostatic sponges. Moreover, curcumin was released in a controlled manner and targeted to accumulate at the surgical site of the tumor, thus effectively inhibiting local tumor recurrence in a subcutaneous postoperative recurrence model (Figure 12A(b)). Comprehensive experiments demonstrated that the e electrospun alginate-targeted drug delivery system could effectively promote wound healing and prevent tumor recurrence.

In addition, the electrospun alginate-targeted drug delivery system can effectively deliver targeted RNA. SA/polyvinyl alcohol/ciprofloxacin wound patches were prepared by electrospinning by Bombin et al. [143]. microRNA-31 (miR-31) and microRNA-132 (miR-132) bound to the positively charged amphipathic peptide RALA to form nanocomplex particles incorporated into the nanofiber wound dressing. MiR-132 was combined with the positively charged amphipathic peptide RALA to form nanocomplex particles incorporated into a nanofiber wound dressing. The composite drug delivery system provided controlled delivery of miRs associated with wound healing, along with good biocompatibility and potent antimicrobial activity. miR-31 and miR-132 expression increased ≥100,000-fold in a mouse fibroblast cell line (NCTC-929) and a human skin keratinocyte cell line (HaCaT). In addition, HaCaT cell migration increased by ≥25% at 4 and 8 h after nanocomplex incubation, and miR-31 and miR-132 target genes were down-regulated. In vivo wound healing experiments in C57BL/6J mice showed that the incorporation of RALA/miR nanoparticles into electrospun nanocomplexes resulted in a significant increase in wound healing rates in the C57BL/6J mice, as compared to the commercial control and the untreated control group (Figure 12B(a)). The wound healing rate increased by more than 60% at day 7 after wound healing (Figure 12B(b)), and the healing speed was significantly increased (Figure 12C), the epidermal thickness increased by ≥1.5, and the number of blood vessels increased by ≥2. The drug delivery system of SA wound dressings with targeted delivery of mRNAs provides a new and advanced strategy for accelerating wound healing and improving the quality of healing tissues.

### 5.6. Other Release

The development of complex platforms for drug delivery has made it possible for composite drug-release modalities. Complex drug delivery systems enable drug delivery platforms with a combination of two release effects. Schoeller et al. [138] deposited sequential layers of CS and SA on electrospun PLGA nanofibers to form polyelectrolyte fiber complexes. Ibuprofen was loaded and exhibited pH-responsive controlled release with a simultaneous biphasic release combination. The drug delivery platform of the composite fiber membrane can bypass the first-pass metabolism of the loaded drug in the liver, which is advantageous for delivering acid degradation-sensitive drugs. Moreover, the biphasic release exhibited by the combined release modality enhanced the therapeutic efficacy of the drug, with the majority of the drug being released within 5 h, followed by sustained release over a 3-day time span.

In addition to complex composite release systems, multistage release modalities with more than two stages have been similarly developed. Kaassis et al. [144] electrospun prepared three-dimensional nanofibrous mats with sodium ibuprofen (SI) crystals embedded in a PEO-SA matrix. A multi-stage release mechanism was achieved in ambient liquid at pH 3 (representative of the pH of the fed state or the stomach of an elderly patient), consisting of an initial burst of release, followed by no further release of the drug for about 120–150 min, and then a final slow release. Adjustment of the SA content altered the rate of retardation in the final stage. Surprisingly, the plateau period was more than 7 h at an SA content of 14.3%, and even after 3 days of release, only about 75% of the embedded drug was released. Chinese medicines can similarly be released in multiple stages by the FF (fragmented fibers)-SA injection drug delivery system [145]. The release of astragaloside IV (AT), the main active ingredient of the Chinese medicine Astragalus and ferulic acid (FA), the main ingredient of *Angelica sinensis*, from the electrospun fibers showed three phases: an initial burst release of about 15% within 24 h, an additional sustained rapid release of 45% within about 1 week, and a gradual release over the following about 3 weeks. The release rate is slightly higher in shorter fragmented fibers.

Composite or multi-stage delivery platforms make it possible to achieve more varied drug release effects to meet the needs of more diverse drug release applications in the biopharmaceutical field.

EADDSs are able to achieve multiple controlled release strategies for drugs by developing different internal structures of single electrospun fibers or by designing multilayered, sparse three-dimensional structures. As shown in Table 2, the loaded drugs are diffused into the external environment by pulsatile release, sustained release, biphasic release, responsive release, targeted release, or other release modes to meet different application scenarios. The loaded drugs include antibiotics, NSAIDs, plant extracts, gas-producing precursors, proteins, growth factors, and ribonucleic acids. However, most studies suffer from a lack of research depth, and cellular, tissue, and biological tests have not been carried out.

**Table 2:** Controlled release effect of different EADDSs and depth of study.

Release Method	Drug-Laden	Controlled-Release Effect	Research Depth	Ref.
Pulse release	Asiaticoside	Released 20% more drug than the control (triterpenoid *Centella asiatica* cream) at the same time over 12 h	Rat experiment: burn wound healing effect is obvious; cytokines and growth factors are positively expressed, promoting the formation of new tissues; pro-inflammatory factors are down-regulated	[84]
	CE	Pulse release of 86% of the drug within 24 h	Antimicrobial test available, no cell or animal test available	[113]
	FA	More than half of the pulse released in 8 h	Cytotoxicity test, non-toxic or only slightly cytotoxic, no animal testing	[114]
	DOX	Full release in 15 min	No cytotoxic effects, no animal testing	[116]
Sustained release	CIP	In vitro release lasts 6 h	With antimicrobial testing, no cell or animal testing	[11]
	H_2_S	Continuous slow controlled release of H2S from the donor scaffold, maintaining a high H2S concentration for at least 21 days	Enhances regenerative capacity of M2 macrophages; attenuates inflammatory polarization of macrophages by reducing intracellular ROS; increases vascular and arterial density in the cardiac border region	[19]
	LEV	90% LEV release extended to 7 days (90% release in 2 days)	Cellular assay: good biocompatibility and non-toxicity. No animal testing	[65]
	Ibuprofen	Sustained release 840 min (rapid release of about 90% of ibuprofen in 150 min without electrospinning)	Cellular assay: showed slight cytotoxicity against L929 cells. No animal testing	[112]
	TCH	Effective release for more than 10 days	With antimicrobial testing, no cell or animal testing	[117]
	KGN	Initial burst release reduced by 8%, sustained slow release achieved over 30 days	Good biocompatibility as demonstrated by the Resazurin assay. No animal testing.	[118]
	CIP	Sustained 14-day release	No antimicrobial, cellular or animal testing	[121]
	Cap	Sustained release over 500 h (21 days)	Cellular assay: showed cytocompatibility with human dermal fibroblasts (HDF); MTT assay: inhibited the proliferation of MCF-7 human mammary cells. No animal testing	[124]
Biphasic release	AF protein	Initial burst release of 6 h, followed by slow release over several days	Cellular assay: no cytotoxicity; contributes to fibroblast adhesion, spreading and proliferation. No animal testing	[115]
	CUR	Initial rapid release, slow release after about 270 min	Cellular assay: good cytocompatibility. No animal testing	[129]
	Insulin	The drug is initially released in very mild bursts and later sustains a slow release for about 10 h	Rat experiment: blood glucose concentration was effectively controlled.	[131]
	CLZ	Approximately 30% of the drug was released by the initial burst within 5 min. a total of 98% of the drug was released after 30 min	Inhibited Candida albicans and Candida dubliniensis; did not have any significant cytotoxic effect on HGF cells. No animal testing	[133]
	pBMP2 + pVEGF	pVEGF was released by rapid diffusion at 3 days, and pBMP2 was released at a sustained slow rate for 21 days	Promotion of in vitro osteogenesis, as evidenced by an increase in calcium deposition and osteogenic gene expression, revealed a significant amount of calcified bone tissue, which gradually matured at 8 weeks	[135]
	GS+ CIP	GS is fully released within 6 days, while CIP can sustain release for up to 3 weeks	Biological experiments have shown that the fiber acts as an excellent promoter of scab removal, blood vessel formation, collagen deposition, and tissue regeneration, with complete healing observed after 21 days	[146]
Response Release	CUR	Cumulative release in an acidic environment (pH 4.2) was 78.13%, much higher than the 22.0% cumulative release at pH 7.4	Characterized antioxidant activity with no antimicrobial, cellular, or animal tests	[15]
	BSA	In vitro simulation of BSA oral-to-colonic release profiles with BSA release in SGF (<4%, 2 h); SIF (15%, 4 h); SCF (75%, 16 h)	Characterized protein conformational stability without antimicrobial, cellular or animal testing	[108]
	Ibuprofen	Faster release in pH neutral than acidic pH (1.0) environments	No antimicrobial, cellular, or animal testing	[138]
Targeted release	CUR	There was no significant burst release and stable release for more than 15 days induced curcumin aggregation at the surgical site of the tumor	Non-toxicity to normal tissues and organs; effective control of curcumin release and induction of curcumin aggregation at the tumor surgical site, thereby inhibiting local tumor recurrence in a subcutaneous postoperative recurrence model	[12]
	HCPT	Intratumoral injection of HCPT-loaded fibrous fragments maintains a high drug concentration within the tumor, which can be released for more than three weeks and attenuates the efflux of HCPT to normal tissue	HCPT has a good spatial distribution within the tumor and has achieved significant results in terms of animal survival, tumor growth inhibition, and tumor cell apoptosis induction	[110]
	microRNA-31 + microRNA-132	The release was greater than 50% in the first four hours, then increased to 80% in 8 h and slowly increased to 84.47 ± 14.09% in 48 h	Downregulation of the target genes of miR-31 and miR-132 increased wound healing rates by more than 60%, epidermal thickness by ≥1.5, and the number of blood vessels by ≥2 at day 7 after wound healing	[143]
Other release	Ibuprofen	pH-sensitive response release and biphasic release. Acidic release was significantly slower at pH 1.0 than at pH 7.4 and pH 5.5. Most of the drug was released within 5 h, followed by sustained release over a 3-day time span	No antimicrobial, cellular, or animal testing	[138]
	SI	Multi-stage release. Initial burst release, followed by no further release of drug for approximately 120–150 min, followed by slow release	No antimicrobial, cellular, or animal testing	[144]
	AT + FA	Three phases: an initial burst release of about 15% over 24 h, an additional sustained rapid release of 45% over about 1 week, followed by a gradual release over about 3 weeks	Significantly enhances neovascularization and blood flow restoration in ischemic tissues, and mouse bioassays show effective promotion of limb preservation with less limb loss	[145]

## 6. Summary 

The strong combination of sodium alginate (SA) and electrostatic spinning has brought into play the biocompatible, gelatinous, and hygroscopic properties of SA while combining the advantages of easy preparation and structural diversity of electrostatic spinning, high specific surface area of nanofiber membranes, high porosity, good mechanical properties, and controllability of drug loading. Electrospun alginate nanofibers (EANFs) are favored for applications in several biomedical fields, such as tissue engineering, regenerative engineering, bioscaffolds, and drug delivery. Although pure SA is not spinnable, and it is impossible to prepare nanofibers containing large amounts of SA, the incorporation of synthetic polymers, such as PEO and PVA, as spinning aids and the modification of SA have greatly improved the spinnability of SA. On the other hand, the non-monofluidic preparation of diverse and novel electrospun fiber structures, such as coaxial, juxtaposed Janus, and tertiary, has also promoted the fiber-forming properties of non-electrospinnable natural polymers.

Electrospun alginate nanofiber systems provide an exceptional platform for drug delivery and controlled release. The electrospun alginate drug delivery system or electrospun-alginate composite drug delivery system realizes a variety of release modes, such as pulsatile release, slow release, biphasic release, stimulus-responsive release, and targeted release. The loaded drugs are mostly antibiotic drugs, and in order to circumvent the abuse hazards of antibiotic drugs and reduce drug resistance, the load of non-zithiomimetic drugs and traditional Chinese medicine extracts has gradually developed. More notably, the EANF system can load and ensure the bioactivity of protein-based, nucleic acid-based, peptide-based drugs and targeted drugs. In addition, they can also be loaded with donors for the indirect delivery of gaseous signaling molecules. In particular, the coaxial structure electrospinning improves the bioavailability and does not hinder the expression of micRNA or signaling factors loaded by the nanofibers. In particular, the drug delivery system in which SA is in the outer layer plays a crucial role in minimizing the erosion of the delivered substance by the non-releasing environment. Moreover, the EANF system showed good non-toxicity and biocompatibility. Overall, the EANF system provides an excellent carrier platform for activity-preserving drug delivery and controlled release.

## 7. Outlook

On the one hand, the emerging multi-fluid fiber structure of electrostatic spinning has not been further applied to a wider range of drug delivery, which needs to be further designed and developed by researchers [147,148,149]. EADDSs enable diverse drug release strategies, but the depth of research on cellular, tissue, and biological assays is insufficient. On the other hand, the combination of EANFs has not been realized for large-scale commercial applications, although it has many advantages in biomedicine and drug delivery. In the near future, controlled delivery and specific release of immune drugs can be developed for efficient and safe immunotherapy of tumors. The delivery of stem cells [150], exosomes [151], virus vectors [152], and photosensitizers [153] by EANFs has to be explored. Overall, the electrospun alginate drug delivery system can realize multiple release modes for drugs and reduce the erosion of non-releasing environments on the delivered substances. Moreover, electrospun fibers protect biological activity, circumvent the harm of drugs to the body, and improve the quality of life of patients with chronic diseases or tumors. Future research can focus on further improving SA spinnability, reducing the proportion of organic solvents, further developing sustainable and green nanofiber materials, and developing electrospun nanofiber materials that can replace petroleum-derived polymers. As for potential commercial electrospun alginate nanofiber-based products, the combinations of electrospinning with other traditional physical and chemical methods [61,154,155,156,157,158] and advanced molecular simulation methods [58,159] may bring out a new atmosphere. 

## Abbreviations

AF	amniotic fluid
Alg	alginate
AT	astragaloside IV
BPB	black phosphorus nanosheet-loaded albumin scaffolds
BSA	bovine serum albumin
CA	cellulose acetate
Cap	capsaicin
CD	cyclodextrin
CD31	cluster of differentiation 31
CE	cardamom extract
CIP	ciprofloxacin
CLZ	clotrimazole
CS	chitosan
CUR	curcumin
DC	direct current
DDW	double distilled water
DMAc	N, N-dimethylacetamide
DOX	doxycycline
EANFs	electrospun alginate nanofibers
EADDS	electrospun alginate drug delivery system
EC	ethyl cellulose
ECM	extracellular matrix
EE	Eudragit® E100
EL	Eudragit® L100-55
ES	Eudragit® S100
FA	folic acid
FKBPL	FK506-binding protein-like
G	α-L-guluronic
GO	graphene oxide
GS	gentamicin sulfate
HCPT	hydroxycamptothecin
HNT	halloysin nanotubes
ID	inner diameter
KET	ketoprofen
KGN	kartogenin
L	levofloxacin
LEV	levofloxacin
M	β-D-mannuronic
MEC	minimum effective concentration
miR	microRNA
MNC	maximum non-toxic concentration
OD	outer diameter
PAR	paracetamol
pBMP2	plasmid DNA encoding bone morphogenetic protein 2
PCL	poly(ε-caprolactone
PDGF-B	platelet-derived growth factor-B
PDLLA	poly (d,l-propyl cross-linker)
PEO	polyethylene oxide
pH	pondus hydrogenii
PLGA	poly(lactic acid-co-hydroxyacetic acid)
PP	poly (d, l-lactic-co-glycolic acid)/polyethylenimine (PLGA/PEI)
PVA	polyvinyl alcohol
pVEGF	plasmid DNA encoding vascular endothelial growth factor
PVP	polyvinylpyrrolidone
RAOA	reductive-amination of oxidized alginate derivative
RNA	ribonucleic acid
SA	sodium alginate
SANF	sodium alginate nanofiber
sCT	salmon calcitonin
SDR	sustained drug release
SEM	scanning electron microscope
SHP	sodium hypophosphite monohydrate
SI	sodium ibuprofen
SSA	sulfated sodium alginate
TCH	tetracycline hydrochloride
TEM	transmission electron microscope
VC	vitamin C
VEGF	vascular endothelial growth factor
WCA	water contact angle
